# The Takotsubo Syndrome: Where the Sympathetic Stress Reflex and the Dive Reflex Conflict

**DOI:** 10.7759/cureus.114143

**Published:** 2026-08-07

**Authors:** John Hayman, Josef Finsterer

**Affiliations:** 1 Clinical Pathology, The University of Melbourne, Melbourne, AUS; 2 Neurology, Krankenanstalt Rudolfstiftung, Vienna, AUT

**Keywords:** bradycardia, carotid chemoreceptors, coronary artery dissecting aneurysm, extracranial internal carotid artery dissection, mammalian diving response, myocardial stunning, pulmonary oedema, sympathetic storming, sympathetic stress response, takotsubo cardiomyopathy

## Abstract

Understanding of the pathophysiology of Takotsubo syndrome (TTS) remains incomplete. This is a hypothesis-driven editorial, proposing a new autonomic mechanism that may explain several important and puzzling features of TTS. Rather than involving a single reflex pathway, we suggest that TTS may result from the interaction and conflict of two evolutionarily conserved autonomic reflex responses.

The first is the sympathetic stress response (SSR), a cardiovascular predominantly sympathomimetic reaction characterised by heightened adrenergic activity, tachycardia and cardiovascular stress. The second is the mammalian dive response (DR), a predominantly parasympathetic reflex characterised by bradycardia, peripheral vasoconstriction, and redistribution of blood flow to vital organs. Usually activated by facial wetting and afferent input through the trigeminal nerve, the DR may be initiated and certainly is moderated from the carotid bodies by way of the glossopharyngeal nerves.

We propose that elements of both the SSR and the DR may be sequentially activated in TTS, producing both augmented and physiologically opposing autonomic effects. This model may help explain several recognised features of the syndrome, including the occurrence of dissecting aneurysms, particularly of the coronary arteries; the prominence of pulmonary congestion and dyspnoea; and the variability in pulse rate, with some patients presenting with bradycardia while others have tachycardia.

Other aspects of the disorder remain unexplained, particularly why some persons are afflicted while others, suffering the same stress, remainsymptom-free. The TTS retains both its mystery and its evocative name.

## Editorial

Takotsubo syndrome: an additional perspective

**Figure 1:**
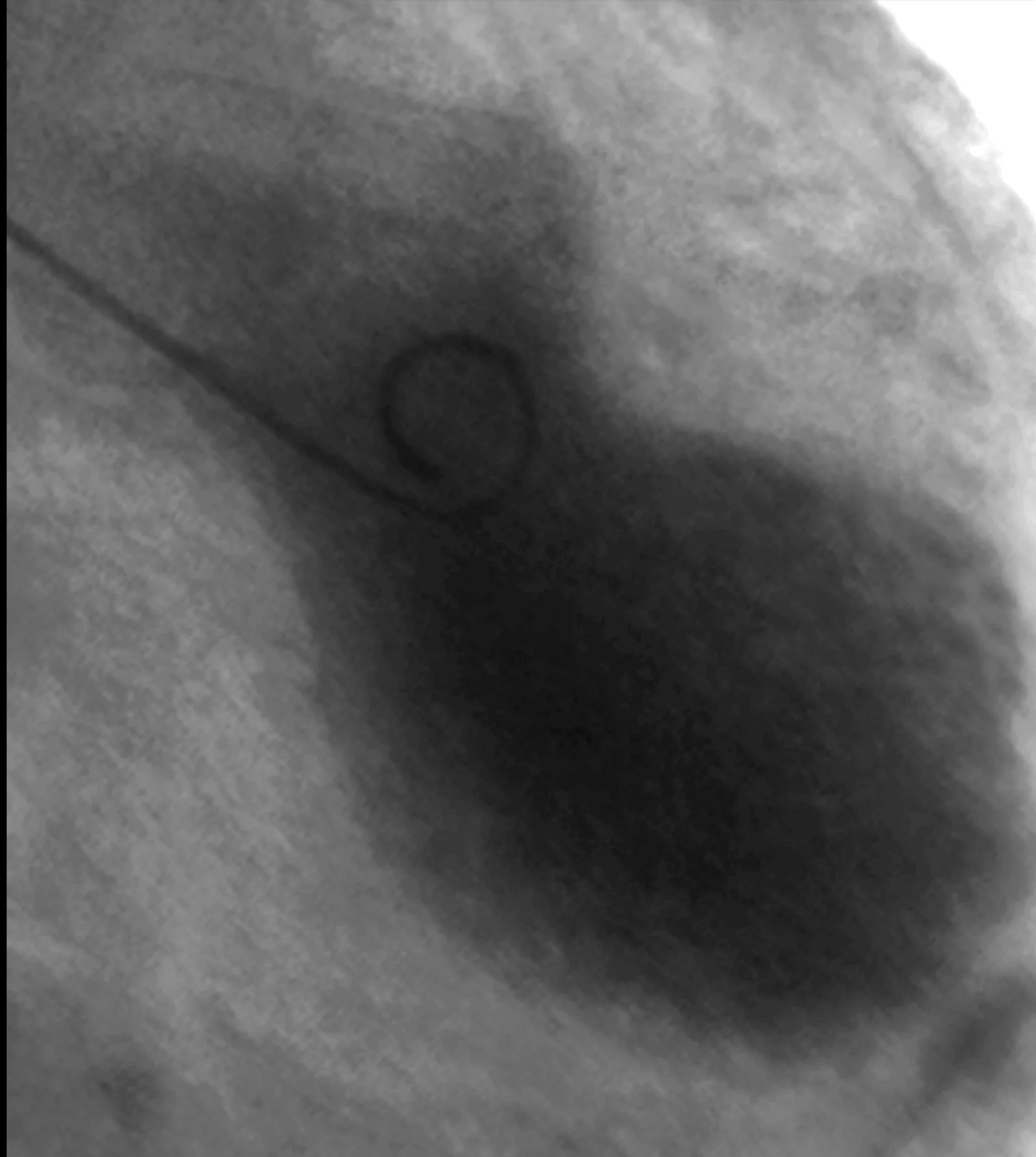
Takotsubo cardiomyopathy, left ventriculogram Image credit: Tara C Gangadhar, Elisabeth Von der Lohe, Stephen G Sawada, and Paul R Helft via Wikimedia Commons. Licensed under CC BY 2.0 (creativecommons.org).

**Figure 2:**
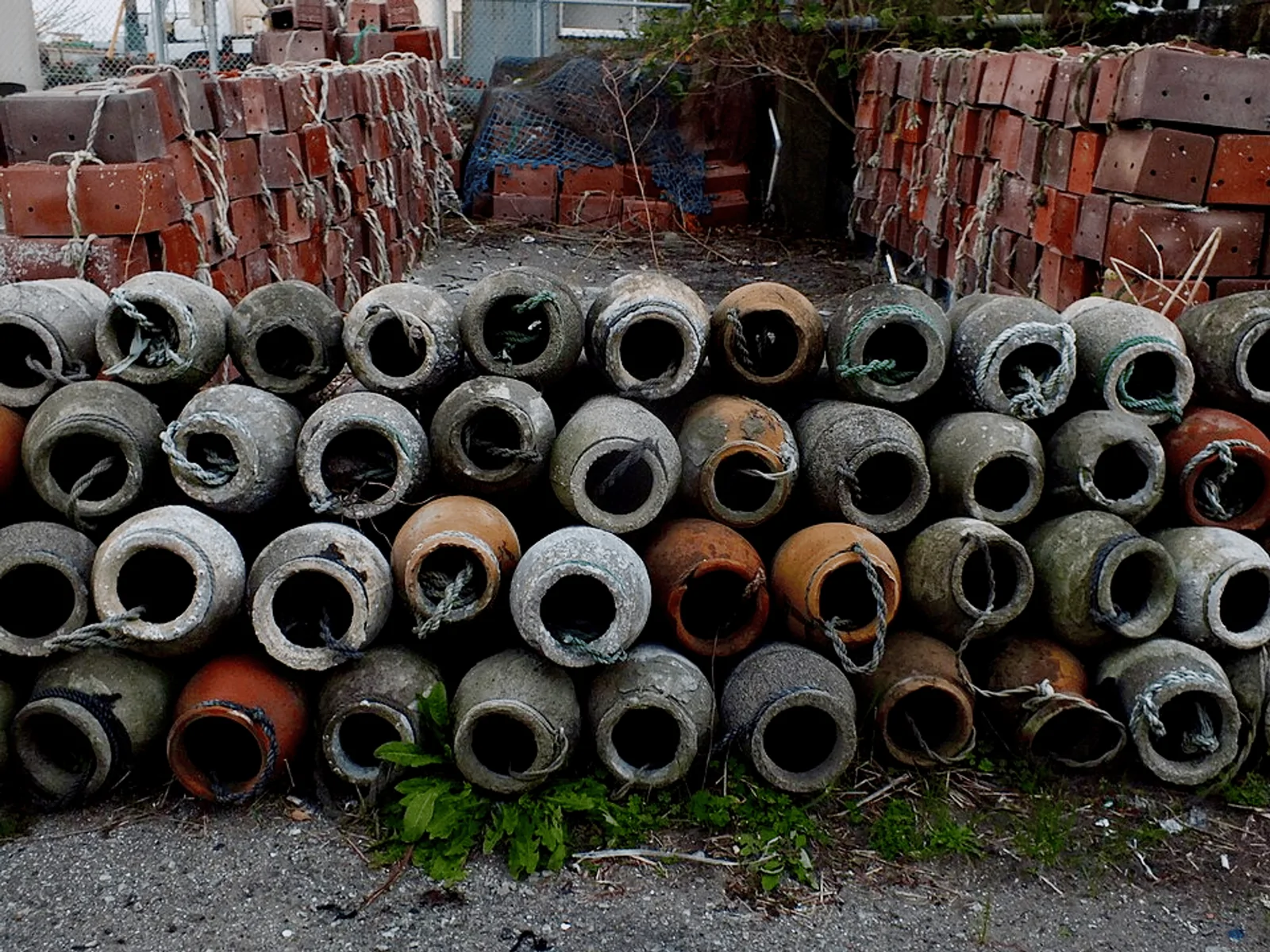
Takotsubo jars, Ohara Fishing Port, Isumi, Japan Image credit: Akiyoshi Matsuoka via Wikimedia Commons. Licensed under CC BY-SA 3.0 (creativecommons.org).

Takotsubo syndrome (TTS), also known as stress cardiomyopathy, myocardial stunning, or broken heart syndrome, was first described in Japan in 1990 by the Japanese cardiologist Hikaru Sato and colleagues [1]. TTS is a non-ischaemic cardiomyopathy characterised by hypokinesis, dyskinesis, or akinesis involving the apical, mid, basal, or all segments of the left ventricle, with hyperkinesis of the not predominantly affected segments, findings classically demonstrated on transthoracic or transesophageal echocardiography or on contrast left ventriculography [2,3]. In up to half of cases the right ventricle can also be involved. The disorder derives its name from the characteristic ventriculographic appearance of the left ventricle in the apical type of TTS, which resembles a traditional Japanese octopus trap (takotsubo) (Figures 1, 2) [1]. TTS occurs worldwide, predominantly in post-menopausal women, and is frequently precipitated by emotional or physical stress, although the trigger may be minor or not identifiable [4]. It may also occur after pleasurable events, hence a further synonym, the 'happy heart syndrome'.

A comprehensive review listed 55 differing emotional or physical events which preceded the episode [2]. Twenty-one cases, all female, occurred in Christchurch, New Zealand, after the earthquake of 2011 [5].

Familial clustering has been reported, including affected siblings and multiple family members [6]. There is also a report involving monozygotic twin sisters who developed TTS five years apart following different emotional stressors [7]. Cases have been reported in association with phaeochromocytoma and the related paraganglioma; both are tumours that secrete catecholamines [8,9].

**Figure 3:**
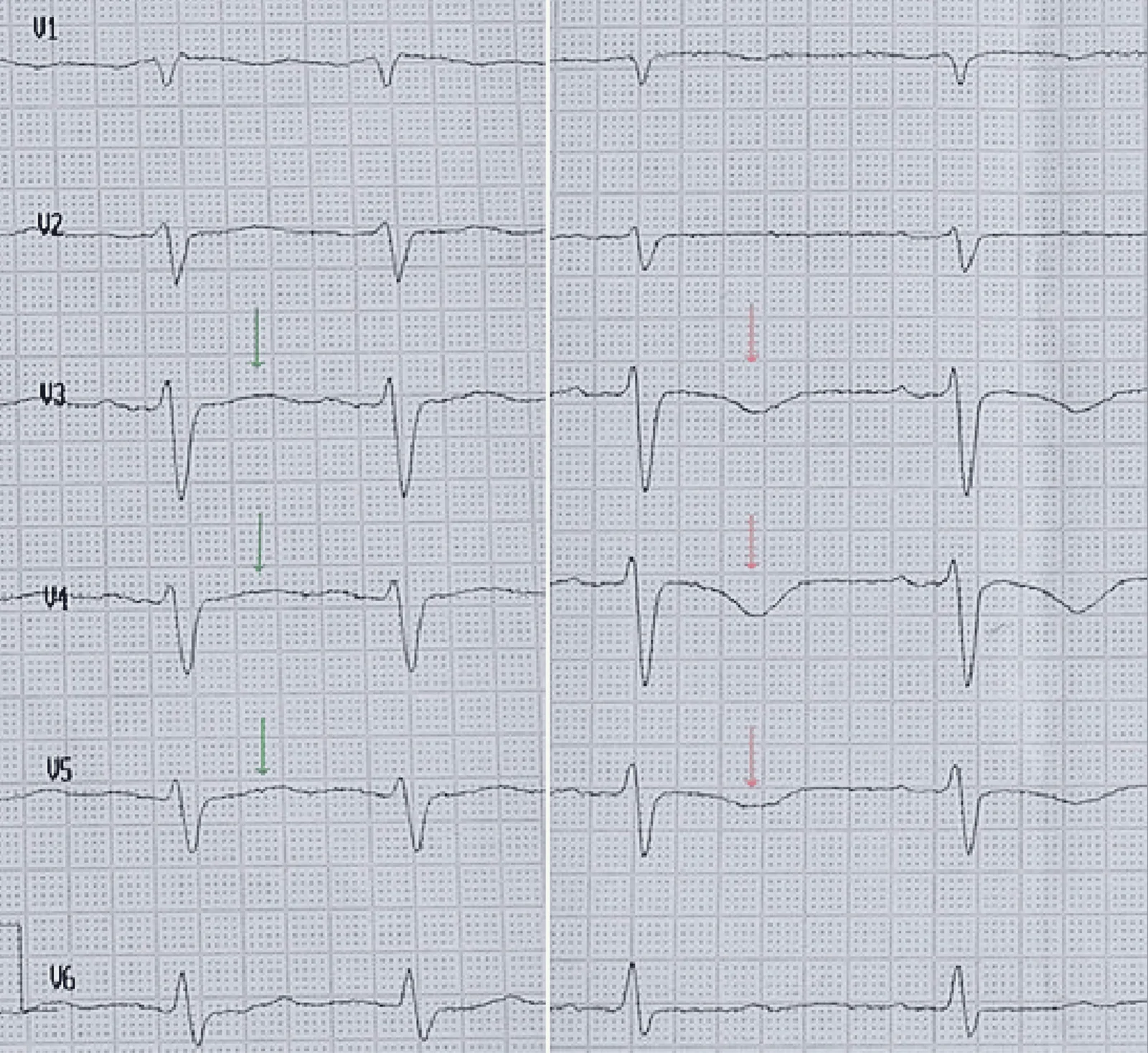
ECG recordings, Takotsubo patient, showing marked bradycardia (33 beats/min) Precordial ECG leads. Left panel: initial ECG; ST segments V3-V5 elevated (green arrows). Right panel: four hours after symptoms; ST changes largely resolved, T waves V3-V5 deeply inverted (red arrows). Image credit: J. Heuser via Wikimedia Commons. Licensed under CC BY-SA 3.0 (creativecommons.org).

Patients commonly present with symptoms suggestive of ST-elevation myocardial infarction (STEMI, or acute coronary syndrome) with chest pain and dyspnoea [10]. Electrocardiographic abnormalities are diverse. Although sinus rhythm with a normal heart rate is more usual, both tachycardia and marked bradycardia are recorded. In one review, ventricular rates ranged from 72 to 155 beats/min [11]. The previously reported monozygotic twins exhibited profound bradycardia, with heart rates of 33 and 29 beats/min respectively [7]. Additional examples include ECGs showing heart rates of 43 beats/min and 50 beats/min in published teaching collections (Figure 3). In addition to sinus bradycardia and sinus tachycardia, TTS can be complicated by complex arrhythmias, such as atrial fibrillation, ventricular arrhythmias, all types of conduction defects, and asystole [12]. 

Creatine kinase, troponin, and brain-natriuretic peptide levels may be elevated. Pulmonary congestion occurs and acute pulmonary oedema has been reported [13]. In contrast to STEMI, coronary angiography does not show significant vessel occlusion. This is an important distinction between TTS and STEMI as clinical and ECG changes may be identical.

Additional complications are acute cardiogenic shock and ventricular wall thrombosis with thromboembolism [12]. Of particular interest is the occurrence of spontaneous coronary artery dissection (SCAD) in association with TTS. Numerous individual cases have been reported, and a review found angiographic evidence of coronary artery dissection in approximately 2.5% of patients diagnosed with TTS [14]. There is a report of one patient who developed symptoms from an internal carotid artery dissection six days after experiencing TTS [15].

Once regarded as a benign and self-limiting condition, TTS is now recognised as a disorder associated with substantial short and long-term morbidity and mortality [10]. In-hospital mortality is comparable to that of STEMI. Long-term follow-up studies have reported all-cause mortality rates of approximately 5.6% per patient-year and major adverse cardiac and cerebrovascular event rates of 9.9% per patient-year. An exception was the cohort of 21 patients affected following the 2011 Christchurch earthquake, all of whom recovered and remained well during follow-up [5].

We propose that TTS results from interaction between two evolutionarily conserved autonomic responses. The initial event is activation of the sympathetic stress reflex (SSR) with catecholamine release and myocardial stunning. The resulting haemodynamic and respiratory consequences subsequently activate components of the mammalian dive response (DR). Sequential activation of these partially antagonistic autonomic pathways may explain several clinical features of TTS that are otherwise difficult to reconcile, including bradycardia, presentation with acute pulmonary oedema, arrhythmias, and an association with arterial dissections.

TTS: a stress-induced cardiomyopathy, with proposed additional features

TTS is a cardiovascular condition which may be induced by psychological as well as physical stress. It differs from acute stress disorder and post-traumatic stress disorder (PTSD), which have psychiatric symptoms and are classified as psychiatric disorders in the Diagnostic and Statistical Manual of Mental Disorders, Fifth Edition (DSM-5) [16]. TTS patients do have a higher incidence of PTSD as well as other stress disorders [17]. In addition, patients are more likely to have other pre-existing psychiatric illnesses such as anxiety, mood disorders, schizophrenia spectrum disorders and neurocognitive disorders [18].

The acute phase of TTS is characterised by activation of the SSR. Central processing appears to involve the locus coeruleus, located in the rostral pons, which is the principal source of cerebral norepinephrine [10]. Through extensive connections with limbic and hypothalamic structures, the locus coeruleus participates in the physiological response to emotional stress. Activation of these pathways stimulates the hypothalamic-pituitary-adrenal axis and promotes rapid release of epinephrine and norepinephrine from the adrenal medulla, producing plasma catecholamine concentrations that may reach two to three times normal levels [19]. The resulting catecholamine surge is thought to produce myocardial stunning through a combination of direct myocardial toxicity, microvascular dysfunction, intracellular calcium overload, oxidative stress, and mitochondrial injury. Myocardial stunning results in damage to the myocardial filaments. Although not a constant feature, this damage commonly results in raised troponin and creatine kinase levels [20].

Current models of TTS largely focus on sympathetic hyperactivation and catecholamine toxicity. However, several clinical features, including bradycardia, peripheral vasoconstriction leading to pulmonary congestion and oedema, and an association with arterial dissections, suggest participation of additional autonomic pathways. These observations raise the possibility that elements of the mammalian DR may be activated concurrently through the SSR, creating a physiological conflict that contributes to the manifestations of TTS.

Mammalian diving response

The mammalian DR is an evolutionarily conserved cardiorespiratory reflex found in all air-breathing vertebrates. In humans it is characterised by apnoea, bradycardia, peripheral vasoconstriction, and an increase in pulse pressure, systolic blood pressure, and asystole [21]. There is contraction of peripheral and splanchnic vessels with increased blood flow to the right ventricle, to the pulmonary circulation, cervical, cerebral, and coronary arteries. The combined effect is preferential preservation of blood flow to the brain and heart during periods of actual or threatened hypoxia, at the expense of less oxygen-sensitive tissues. The vasoconstriction of selected vessels serves to reduce the systemic lactic acidosis that would otherwise occur with the return of blood from hypoxic areas.

The DR is most active in the first year of life [22]. Infants develop apnoea or bradycardia/asystole with simple blowing on the face. Older children respond with facial immersion or wetting of the face. Apnoea is part of this response. Voluntary apnoea serves both to initiate the response and to increase the depth.

Although of survival value in an aqueous environment, the DR may have adverse consequences. Although definitive proof is lacking, activation of the DR has been proposed as a contributing factor in sudden infant death syndrome (SIDS), sudden unexpected death in epilepsy (SUDEP), immersion pulmonary oedema (IPO), and arterial dissections occurring in relation to aquatic activities [23].

Dissecting aneurysm may represent a potential marker of previous activation of the DR. The increased systolic and pulse pressures result hemodynamically in increased pulsatile distension of aorta and arteries with subsequent elastic fibre degeneration and potential dissection along a weakened media [24]. This elastic fibre degeneration was observed in an early case report [25]. As well as head, neck arteries and aorta, a dissecting aneurysm has been reported of a coronary artery in a scuba diver [26]. Reports of the occasional aortic dissection have been recorded in surface swimmers. One case of dissecting aneurysm has occurred with IPO. It has also been reported in association with SUDEP and also with non-fatal epilepsy [27]. Although varied in their clinical associations, these cases of dissection aneurysm are all considered to be the sequel of an activated DR with high systolic and pulse pressures.

Activation of the mammalian diving response

The DR may be initiated by stimulation of trigeminal nerve endings in the face and nasal passages during facial immersion or wetting [28]. Its intensity is modulated by inputs from the carotid and aortic chemoreceptors [29]. When arterial oxygen tension falls below a critical threshold, 60 mm Hg in a healthy individual, afferent signals from the carotid bodies travel via the glossopharyngeal nerve to the brainstem. Stimulation of the carotid bodies or stimulation of the glossopharyngeal nerve in the seal results in bradycardia [30]. In addition to responding to changes in blood gases, the carotid bodies are sensitive to several circulating hormones, including catecholamines [31]. With the DR, efferent autonomic output from the brainstem produces intense peripheral vasoconstriction while simultaneously preserving cerebral and coronary perfusion. Experimental evidence suggests that trigeminal and carotid body inputs act synergistically to produce the full expression of the DR [32]. The augmented blood return from the periphery following an activated DR further increases pulmonary blood flow with the potential to result in pulmonary congestion and pulmonary oedema.

Concept development

One of us (JH) in 1986 reviewed an autopsy on a previously healthy 20-year-old scuba diver who died from a ruptured aortic dissecting aneurysm [25]. This was originally thought to be an isolated event but a further 18 cases of aortic and head and neck artery dissection were subsequently reported and one case of coronary artery dissection, all in scuba divers [32]. The DR was implicated in this and, as well, in a variety of adverse events, some well away from water [23]. One reference linked dissecting aneurysm of the internal carotid artery with TTS [15] and there are numerous reported cases of dissection of the coronary arteries with TTS [14]. Apart from dissections, other aspects of TTS also appear to involve the DR.

The coronary artery dissections were taken as evidence of an active DR; the one report of dissection of the internal carotid artery indicated that the coronary artery dissections were not simply a sequel to myocardial dyskinesia [15].

Autonomic conflict

Autonomic conflict is not a new concept. Interaction between the DR and PTSD has previously been proposed [33]. The physiological reactions induced by the DR include slowing of the heart rate, conservation of oxygen, and conservation of energy, while the fight or flight response, characterised by PTSD, has opposite physiological effects. Therapeutically induced forms of the DR are used to treat symptoms of PTSD. There is overlap in patients experiencing TTS and PTSD, and PTSD may follow an episode of TTS [17]. 

Autonomic conflict has also been proposed between the powerful antagonistic ‘cold shock response’ and the DR [34]. The former involves the activation of an SSR-driven tachycardia while the latter promotes a DR-mediated bradycardia. Bradycardia vs tachycardia promotion may account for the arrhythmias that follow cold water immersion in healthy volunteers.

Susceptibility

The inheritance of susceptibility to TTS may be similar to that now accepted for many psychiatric disorders, including PTSD [35]. These are now considered to be due to the combined effects of hundreds if not thousands of genes, each gene contributing a minuscule amount to the overall risk factor. There is shared genetic risk across psychiatric disorders with disorders such as schizophrenia occurring in one sibling and bipolar in another. It is a plausible proposal that genes involved in psychiatric illnesses are some of many genes that result in a susceptibility to TTS [36]. This provides an explanation for the associations between TTS and psychiatric illness; TTS and psychiatric illnesses have susceptibility genes in common.

Proposed pathophysiology

A provisional proposed pathophysiology for TTS is as follows: firstly, the ‘fight or flight’ SSR is triggered by impulses through the hypothalamic-pituitary-adrenal axis. An adrenal storm results in myocardial dyskinesia; this also produces changes in sensitivity in the carotid bodies [29]. Cardiac output is reduced by decreased stroke volume and reduced ejection fraction with lowering of arterial O2 saturation, activating the carotid bodies, and initiating a form of DR.

Parasympathetic impulses, acting through the vagal nerve, slow down the heart rate, at times resulting in bradycardia. Systemic hypertension, high pulse pressure and the bradycardia, all part of the DR in humans, result in haemodynamic stress in coronary, head and neck arteries. Dissection of these vessels is seen as a consequence of this stress.

Activation of the SSR, followed by a correctional, predominantly parasympathetic DR-related response, provides a plausible if unproven explanation for TTS, consistent with the clinical, radiological and ECG features. Bradycardia vs tachycardia, conflicting SSR and predominantly parasympathetic DR may result in an irregular pulse with frequent ectopic beats, a similar situation to that proposed for the ‘cold shock response’ and the DR [34].

Conclusions

This editorial gives a suggested explanation as to why some TTS patients record bradycardia, why some develop dissecting aneurysms and why pulmonary congestion and oedema may be prominent features in TTS. Activation of the DR is proposed for these clinical characteristics. A multigenetic genome with hundreds of genes shared with those susceptible to psychiatric disorders provides an explanation as to why psychiatric disorders are more prevalent in TTS patients.

This editorial should be seen as being hypothesis-generated. It is subject to testing by measurement of sympathomimetic hormone levels and by responses to therapy. Adrenergic antagonists may be indicated if there is tachycardia; anticholiergics if there is bradycardia. 

This review does not address the question as to how stress, even unapparent stress, results in the TTS. It does not explain why it occurs predominantly in females [35], nor does it explain why TTS persists long after any stressful event has dissipated. Answers may lie in our long distant evolutionary past and in our present complex variable genotypes.
